# A Policy for Optimizing Sub-Band Selection Sequences in Wideband Spectrum Sensing

**DOI:** 10.3390/s19194090

**Published:** 2019-09-21

**Authors:** Yangyi Chen, Shaojing Su, Junyu Wei

**Affiliations:** College of Intelligent Science and Technology, National University of Defense Technology, Changsha 410073, China; chenyangyi09@nudt.edu.cn (Y.C.); susj-5@163.com (S.S.)

**Keywords:** sub-band selection, non-stationary multi-armed bandit, wideband spectrum sensing

## Abstract

With the development of wireless communication technology, cognitive radio needs to solve the spectrum sensing problem of wideband wireless signals. Due to performance limitation of electronic components, it is difficult to complete spectrum sensing of wideband wireless signals at once. Therefore, it is required that the wideband wireless signal has to be split into a set of sub-bands before the further signal processing. However, the sequence of sub-band perception has become one of the important factors, which deeply-impact wideband spectrum sensing performance. In this paper, we develop a novel approach for sub-band selection through the non-stationary multi-arm bandit (NS-MAB) model. This approach is based on a well-known order optimal policy for NS-MAB mode called discounted upper confidence bound (D-UCB) policy. In this paper, according to different application requirements, various discount functions and exploration bonuses of D-UCB are designed, which are taken as the parameters of the policy proposed in this paper. Our simulation result demonstrates that the proposed policy can provide lower cumulative regret than other existing state-of-the-art policies for sub-band selection of wideband spectrum sensing.

## 1. Introduction

With the rapid development of internet of things and 5G technologies, wireless spectrum resources are becoming scarcer. Cognitive radio, as a promising solution for efficient radio spectrum utilization [1,2], meets many challenges, especially spectrum sensing technology [3], wideband spectrum sensing is one of challenges. Various solutions have been proposed on wideband spectrum sensing, such as the single-channel sub-Nyquist sampling algorithm [4] and compressed sensing [5]. Although compressed sensing is the most promising method, it is still difficult to solve the spectrum sensing problem for wideband wireless signals when faced with noncontiguous bands, complex, and changeable electromagnetic environment [6]. Therefore, the research on traditional wideband spectrum sensing architecture is still meaningful: Segment the wideband spectrum into a set of sub-bands according to the requirement of users, and then conduct spectrum sensing for a certain sub-band at each time slot [7]. In the case of fixed spectrum sensing algorithm, how to determine the sensing sequence of sub-bands is the main research content to improve the sensing ability of cognitive radio.

In the traditional wideband spectrum sensing architecture, the spectrum sensing method of sub-band includes narrow band spectrum sensing algorithms and wide-band spectrum sensing algorithms, so the spectrum sensing algorithm can be selected according to the application requirements [8,9,10]. Sub-band selection problem is a kind of optimization problem, in the study of spectrum sensing there are many optimization problems: Energy efficiency optimization [11], sub-channel allocation optimization [11], and throughput balancing [1], so the sub-band selection problem can be modeled as a exploration and exploitation tradeoff problem as well as a spectrum dynamic allocation problem [12]. 

Currently, multi-armed bandit (MAB) mode has been employed to analyze the spectrum dynamic allocation problem. The dynamic rule, which is governing the selection of the machine to be played, is called a policy, it is commonly denoted by π. The MAB problems can be divided into stationary MAB problems and non-stationary MAB (NS-MAB) problems [13]. In this paper, due to the dynamic nature of the primary user’s signals in sub-bands, the sub-band selection problem is generally modeled as a NS-MAB problem. Much attention has been devoted to NS-MAB problems, and some policies have been proposed, such as: Discounted upper confidence bound (D-UCB) policy and sliding window upper confidence bound (SW-UCB) policy. To the author’s knowledge, because of the uncertainty of the change, these policies have poor performance in solving the sub-band selection problem.

Therefore, we propose an optimized D-UCB (O-D-UCB) policy to improve the performance of the decision strategy on sub-band selection by optimizing the discount factor and exploration bonus in the D-UCB algorithm. According to multiple experimental results, it is shown that the performance improvement of the policy proposed in this paper is verified. The main contributions of this paper are:

(1). Several new discount functions and exploration bonuses which have been designed for the O-D-UCB policy;
(2). The effect of discount function and exploration bonus on the O-D-UCB policy experimentally studied;
(3). An O-D-UCB policy for sub-band selection problems is proposed and its performance is compared with other policies through simulation experiments.

The remainder of the paper is organized as follows. In Section 2, related works are reviewed. In Section 3, the NS-MAB problem is modeled and analyzed, and we propose a policy based on D-UCB in Section 4 a series of discount functions and exploration bonuses are employed. In Section 5, we designed multiple experiments to verify the influence of different discount functions and exploration bonus on D-UCB policies, and compare our policy with other policies. In Section 6, we briefly discuss the experimental results. The paper is concluded in Section 7.

## 2. Related Works

**Figure 1:**
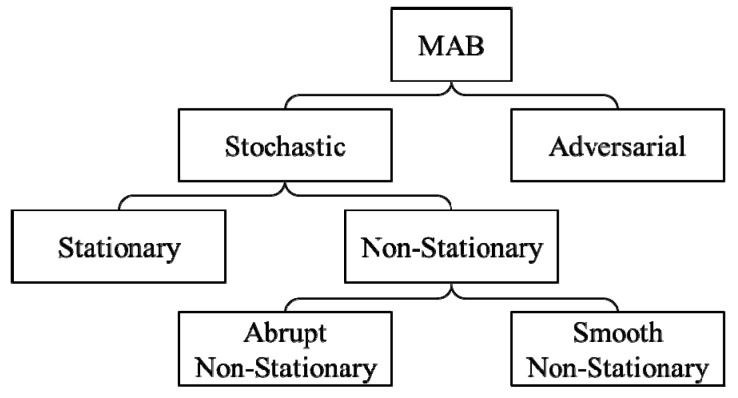
The taxonomy of multi-armed bandit (MAB) mode.

The modeling of the exploration-exploitation tradeoff problem with MAB was originally proposed in the context of drug testing in [14], and placed in a general setting in [15]. Since its inception, MAB mode with various modifications has been studied, and the taxonomy of MAB modes is summarized in Figure 1 which based on the reward model. In a stochastic MAB mode, the rewards are generated from stochastic distributions, but in an adversarial MAB mode, the rewards are generated by a process that cannot be treated as a stochastic distribution [16].

### 2.1. Stochastic Stationary MAB

If the stochastic distributions of rewards are stationary, it will be a stationary MAB mode. To address this question, many researchers have proposed a series of methods including: Randomized, UCB-based, recency-based, and deterministic.

The simplest policy is randomized policy, the most typical of which is the greedy policy, the core idea of the greedy policy is to selects the arm with the highest expected reward at a time. Hence there is no exploration in the greedy policy. In order to balance exploration and exploitation, Watkins proposed the ε-greedy policy [17], in which, explores has a probability of ε and exploits has a probability of 1-ε, where ε is close to zero. On this basis, several improvements of this policy appear: ε-first [18], ε-decrease [19], greedy mix [20,21], and least taken [21]. Another randomized policy is soft-max policy which was proposed by Luce in [22], and it is a variant of the ε-greedy policy. When exploring, instead of randomly selecting the arm, the policy selects the arm with maximum probability which is calculated by soft-max function. It can be transformed into decreasing soft-max policy [19] and EXP3 cluster policies [23,24]. 

The UCB policy is the typical representation of UCB-based policies [25,26]. In this policy, we can measure the potential optimal value by an upper confidence bound of the reward value (it also can be called exploration bonus). In [26], a class of policies based on sample means and upper confidence bounds were proposed, and the policy was further developed in [27] by improving a constant in the UCB policy. Recently the KL-UCB policy [28] and Lin-UCB policy [29] were proposed to extend the application scenario of MAB mode.

The recency-based policies and deterministic policies are mainly used in Markovian reward environments, and the main representatives are the RBE policy [30], RBERC policy [12], and DSEE policy [31]. In addition, there are other new policies for solving stochastic stationary MAB problems, such as: [21,32,33,34].

### 2.2. Stochastic Non-Stationary MAB

If the stochastic distributions of rewards are not stationary, it will be a NS-MAB mode. Further, we can divide it into smooth NS-MAB and abrupt NS-MAB which are based on whether the change of rewards are continuous or not; the abrupt NS-MAB mode is widely used and studied.

NS-MAB mode is the extension of stationary MAB mode, and the NS-MAB mode is more realistic in some scenarios. Following the distinction in [35], there are two main approaches to deal with NS-MAB. One is the passively adaptive policy which is unawares the change of reward and updates its decision just based on the observations. The other one is the actively adaptive policy which monitors the environment for getting change points and updates their decision based on the change points. ε-greedy policy is the simplest passively adaptive policy, and the EXP3.S policy is a variant of the ε-greedy policy which can get better performance in NS-MAB problems [36]. In addition, there are a number of UCB cluster policies for NS-MAB which are based on UCB policy of stationary MAB. 

The discounted UCB (D-UCB) policies were proposed by Kocsis in [37], and other studies have refined these policies [38,39]. The D-UCB policy averages past rewards with a discount factor which gives more weight to recent plays. The sliding window UCB (SW-UCB) policy was also proposed in [39] which relies on a local empirical average of the observed rewards, using only the last plays, τ. Based on these, non-stationary recency-based exploration (NRBE) policy and windowed recency-based exploration (WRBE) policy were proposed in [40], these polices are the combination of UCB and RBE. Besides, the periodic version of the tuned UCB policy was proposed in [41] which regularly reset, always, after a fixed number of sensing instances. The previous policies for MAB mode are summarized in Table 1.

## 3. System Model

### 3.1. NS-MAB Model

In this paper, we modeled the sub-band selection problem as an abrupt NS-MAB problem.

**Definition** **1.** *A NS-MAB problem is a tuple*〈K,ℛ〉*where*:

- K={1,2,....,K}*is a set of arms;*
- ℛ:=R(r|k,t)*is an unknown probability distribution of reward r given the chosen arm k at time t, where*$\exists k\in K,t_{1},t_{2}$*s.t.*$R(r|k,t_{1})\neq R(r|k,t_{2})$.

We denote with t={1,2,....,n} be the sequence of sense slots. The smaller the *t* value, the more recent the samples are. $X_{t}^{k}\in [0,1]$ is the reward of arm *k* at slot *t*. The rewards ${\left\{X_{t}^{k}\right\}}_{t\geq 1}$ of arm *k* are modeled as a sequence of independent random variables from potentially different distributions unknown to sensor node, and the expectation of reward is denoted by $\mu_{t}(k)=E\left[X_{t}^{k}\right]$. We define the optimal arms as $i^{*}=\arg max_{k\in K}\mu_{t}(k)$, and the optimal reward is $\mu_{t}(i^{*})$. In NS-MAB, there is not only an optimal arm $i^{*}$ since the distributions change, so we denote $i_{t}^{*}$ to be the optimal arm of each time step *t*. We define $\Delta =(\mu_{t}(i_{t}^{*})-\mu_{t}(i_{t}))$ as the difference in terms of expected reward which called is regret, where $i_{t}$ is the arm selected by policy in time step *t*.

**Definition** **2.** *The cumulative pseudo-regret of policy*π*on a NS-MAB problem is defined by Equation (1), where the T is the size of time slot*.

(1) $$R_{T}=\sum_{t=1}^{T}\left(\mu_{t}(i_{t}^{*})-\mu_{t}(i_{t})\right)$$

In the policy π, if the optimal arm is selected at each time slot, the cumulative pseudo-regret will be zero: $R_{T}=0$; if the selected arm away from the optimal arm, the cumulative pseudo-regret will be much greater than zero. Consequently, the cumulative pseudo-regret can reflect the quality of the policy.

### 3.2. Sub-Band Selection Problem

We use the traditional wideband spectrum sensing architecture in this paper. We should segment the spectrum into a set of sub-bands according to the requirement of users, and then conduct spectrum sensing for a certain sub-band at each time slot, in the traditional sensing architecture. As we discussed in the previous section, the sensing sequence of sub-bands is an important factor of sensing performance of wideband spectrum sensing.

In this paper, we mainly focused on the sub-band selection problem. We divide the wideband signal into multiple sub-bands according to channel division, where each sub-band is a channel. We assume that the wideband signal can be divided into *N_b_* sub-bands (channels). In the absence of prior knowledge of the channel (sub-band), assume that the channel obeys the binomial distribution. In this model, there are two state of the channel: $H_{0}$, meaning that only the noise (no primary user’s signal) exists and $H_{1}$, meaning that both the PU signal and the noise exist. We set that the value of state $H_{0}$ is zero ($V_{H_{0}}=0$) and the probability of state $H_{0}$ is $p_{H_{0}}$; the value of state $H_{1}$ is one ($V_{H_{1}}=1$) and the probability of state $H_{1}$ is $p_{H_{1}}$, where $p_{H_{0}}+p_{H_{1}}=1$. So, in the NS-MAB model, the channel can be modeled as the arms, and the state of the channel can be modeled as the reward of the arm.

**Definition** **3.** *The reward of arms in the NS-MAB model is defined as the expectation of the binomial distribution of channel:*

(2) $$E_{s}=V_{H_{0}}*p_{H_{0}}+V_{H_{1}}*p_{H_{1}}=p_{H_{1}}$$

Because of the dynamic nature of the channel, the probability distribution of binomial distribution may change over time. So in this paper, by analyzing the variation law of the channel state probability distribution function, we summarize four typical scenarios: Scenario 1: The probability distribution functions of all channels remain stable; scenario 2: Only one channel’s probability distribution function changes, and the rest remain stable; scenario 3: The probability distribution function of multiple channels changes simultaneously, and the others remain stable; scenario 4: The probability distribution function of multiple channels changes at different times. 

Among the four scenarios, scenario 1 is the simplest, and multiple assumptions are made on the basis of the practical situation of electromagnetic environment. Scenario 4 is the most complex, which is the most consistent with the practical situation of electromagnetic environment, and the assumptions are the least. But these four scenarios need to make the following assumptions: When the probability distribution function change, it will not change again in *M* time slot.

## 4. Methods

### 4.1. Algorithm

On the basis of D-UCB policies [12,39,40], it is optimized by adding discount function and redesigning exploration reward to make it more suitable for the complex and changeable electromagnetic environment. The optimized D-UCB policy is described in the Algorithm 1.

**Algorithm 1** Optimized D-UCB (O-D-UCB) Algorithm **Input:** Discount function(f(x)), exploration bonus (B(x)), and sampled data($X^{k}(s)$)**Output:** The state of the channel perceived by the spectrum sensing algorithm (Cs)1:Initialization, sense each band once.2:**for each**t=K+1,K+2,⋯:3:  **Set**
$U_{k}(t)=X_{k}(t)+B_{k}(t)$;4:  Draw action $i_{t}$, $i_{t}=\arg\max U_{k}(t)$
5:  Receive reward $X_{k}(t)\in [0,1]$6:  **for all**
k=1,…,K set:7:   $X_{k}(t)=\sum_{s=1}^{t}f(s)X^{k}(s)1_{i_{s}=k}/N_{k}(t)$
8:   
$N_{k}(t)=\sum_{s=1}^{t}f(s)1_{i_{s}=k}$
9:
   
$B_{k}(t)=B(t)$


In Algorithm 1, the indicator function $1_{u}$ obtains value 1 if *u* is true and otherwise 0. We can know from Algorithm 1 that the core index of the O-D-UCB policy selection arm is $U_{k}(t)$ which is composed by $X_{k}(t)$ and $B_{k}(t)$, and it can be written as follows:

(3) $$U_{k}(t)=X_{k}(t)+B_{k}(t)$$

where $X_{k}(t)$ is employed to estimate the exploitation by discounted averages, and $B_{k}(t)$ is exploration bonus which represents the exploration. The calculation expressions of $X_{k}(t)$ and $B_{k}(t)$ are shown in the Algorithm 1 In [39], the power function is used as the discount function which described as $f(x)=\gamma^{x}$, then the $X_{k}(t)$ can be written as follows:

(4) $$X_{k}(t)=\sum_{s=1}^{t}\gamma^{t-s}X_{t}^{k}1_{i_{s}=i}/\sum_{s=1}^{t}\gamma^{t-s}1_{i_{s}=i}$$

The exploration bonus in [39] is shown as follows, where $N_{k}(t)=\sum_{s=1}^{t}\gamma^{t-s}1_{i_{s}=i}$ and ξ is the bias parameter:

(5) $$B_{k}(t)=2B\sqrt{\xi (\log\sum_{i=1}^{K}N_{i}(t))/N_{k}(t)}$$

### 4.2. Parameters

#### 4.2.1. Discount Function

The discount function is designed to weight data; the recent samples have higher weight and more representatives of changes in the distribution of reward. Based on this, the monotonic decreasing function can be designed as a discount function of O-D-UCB policy. Therefore, we designed a variety of discount functions; the O-D-UCB policy can choose the appropriate discount function according to different application scenarios. 

The exponential function can be used to design a discount function whose expression is $f(x)=\gamma^{x}$. The base of exponential function determines the properties of the exponential function. If 0<γ<1, it is a concave function with monotonic decreasing; when γ>1, it is a concave function with monotonic increasing, and if γ is bigger or smaller than 1, the curve is going to be very steep, the value of the function is going to change dramatically. Therefore, when the exponential function is used as the discount function, the base (γ) should be close to 1, otherwise the historical samples cannot be fully utilized, as in [39], the base (γ) is set as γ=0.9982.

**Figure 2:**
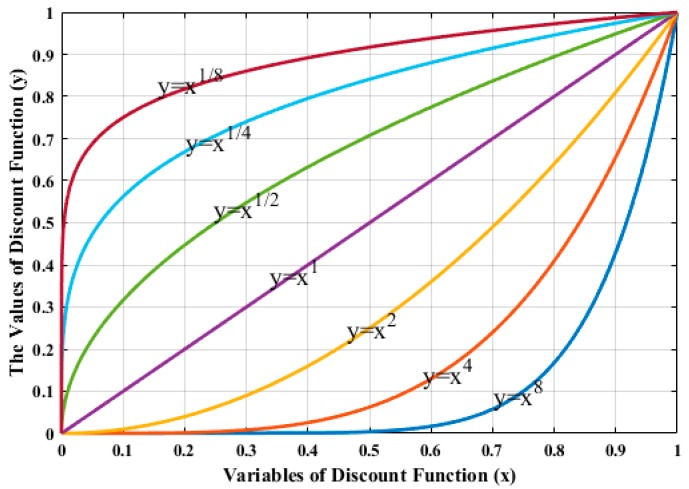
Power function with different exponents.

Besides, we can design a discount function based on a power function and the expression of the power function is $f(x)=x^{a}$. Similar to the exponential function, the exponent (a) determines the properties of power function. If 0<a<1, it is a monotony increase by degrees convex function, when a>1, it is a monotony increase by degrees concave function, and when a=1, it is a linear function, and the function curves with different exponents is described in Figure 2. 

As we can know from Figure 2, the power function is monotonic increasing function, which is inconsistent with the monotonic decreasing function of discount function. Therefore, when using it to design discount function, we need to redesign the variables of power function. In this paper, we modified the expression of the power function, and it is shown as follows:

(6) $$f(x)={\left((N-x)/N\right)}^{a}$$

In addition, window function can also be used to design discount function. Such as the SW-UCB policy which is proposed in [39], only the τ most recent samples are considered in computing its mean reward. The rectangle window employed by [39], seeing from another angle, it is another type of discount function. It means that the weight of the τ most recent samples is 1 and the weight of remaining samples is 0, and the expression is shown in (7). Therefore, we can adjust the window function to design different discount functions for the O-D-UCB policy.

(7) $$R_{\tau }(x)=\left\{ \begin{array}{l} \begin{array}{cc} 1, & 0<x<\tau ; \end{array}\\ \begin{array}{cc} 0, & x>\tau . \end{array} \end{array}\right.$$

Many window functions are proposed in the process of filter design. By analyzing the characteristics of various window functions, we designed the discount function based on the Hanning window. The expression of the improved Hanning window function is described as follows:

(8) f(x)=(1+cos(pi∗x/N))/2

**Figure 3:**
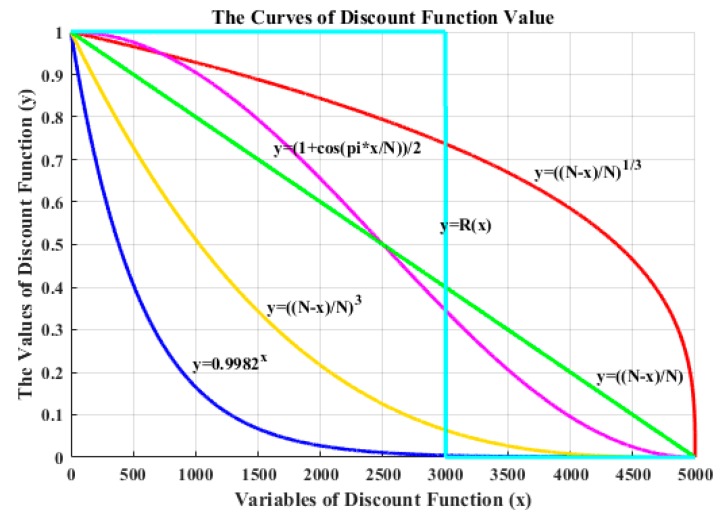
Discount functions studied, and the expression of the function are labeled on the curve.

In this paper, six discount functions are designed by the above three types of functions with different parameters. The function curves and expressions are shown in Figure 3. 

When verifying the impact of the discount function on the performance of the policy, we should fix the exploration bonus; the expression of the exploration bonus is shown in Equation (5). Based on the discount functions shown in Figure 3, we designed six different D-UCB policies. 

We set that the UCB-E stands for traditional D-UCB policy using exponential function as discount function; the UCB-L means that the discount function of the D-UCB policy is a linear function (the exponent of the power function is 1); the UCB-P-1/3 indicates that the discount function of the D-UCB policy is a power function with an exponent of 1/3; the UCB-P-3 represents the discount function of the D-UCB policy as a power function with an exponent of 3; the D-UCB policy with the improved Hanning window as its discount function is called UCB-W-H; and the UCB-W-R policy is a D-UCB policy with the discount function is rectangular window. 

The correspondence between the abbreviation of policy and the discount function expression is as shown in the Table 2. Different discount functions reflect the degree of utilization of the sample by the policy; in different application scenarios, policy needs to have different degrees of utilization of the samples. Therefore, we need to determine the discount function that best fits the application requirements before the simulation experiments.

#### 4.2.2. Exploration Bonus

The exploration bonus is designed to promote the sensing of channel which has not sensed for a long time. By analyzing the characteristics of different exploration bonus under stationary condition, it was found that those exploration bonuses are generally designed with the average reward of the arm as the independent variable, and they are generally concave functions with monotonic decreasing. This is also supported by intuition: When the average reward difference of arms is small, it is necessary to increase the exploration bonus fast in order to find the optimal arms; when the average reward difference of arms is large, the exploration bonus should be slowed down in order to inhibit excessive exploration.

In non-stationary problems, the exploration bonus should be modified to ensure that explored the optimal channel at once when the rewards change. In general case, the exploration bonus of non-stationary reward can be written as Equation (5), where the parameter ξ can be adjusted by requirements. 

Here, we introduce another exploration bonus to solve NS-MAB problems, while it performs well in stationary conditions which is shown as follows:

(9) $$B(x)=\xi \sqrt{\mathrm{var}(x)/N_{k}(t)}$$

where $\mathrm{var}(x)=X_{k}(t)(1-X_{k}(t))=X_{k}(t)-X_{k}{\left(t\right)}^{2}$ is the statistical variance of each arm reward, ξ is the bias parameter, it is noted that ξ=1.96 through theoretical analysis in stationary conditions [42]. In non-stationary conditions, since the sample values are weighted, the bias parameters should be adjusted with discount functions. 

The exploration bonus based on statistical variance is also supported by intuition: When the variance of arm reward is large, it means that the arm has not been fully explored and further exploration is needed. When the variance of arm reward is small, it means that the arm has been fully explored and no more exploration is needed.

It is the same with discount function that we should fix the discount function when studying the impact of exploration bonus on policy performance. In this paper, we fix the discount function as:

(10) $$f(x)=\gamma^{x}$$

where γ=0.9982. Based on the previous research, we designed two kinds of exploration bonus during the experiment. EB-a represents the exploration bonus based on the traditional D-UCB policy, and its expression is Equation (5); EB-b represents the exploration bonus based on statistic variance, and its expression is Equation (9).

Different exploration bonus represents the degree to which sensing strategies attach importance to exploration, and also reflect the balance between exploitation and exploration of strategies. Therefore, it is necessary to choose the exploration bonus reasonably. When the rewards of arms change frequently, the intensity of exploration should be increased; otherwise, the exploration effort should be reduced.

In this paper, in order to get the best performance, we should design experiments to find the optimal exploration bonus before the simulation experiments.

## 5. Experiments

In this paper, we adopt the traditional wideband spectrum sensing architecture, and we assumed that the sub-band spectrum sensing method as shown in literature [10] which is based on multi-resolution singular spectrum entropy; the sub-band selection algorithm which is proposed in this paper was used to determine the sub-band sequence. Therefore, each sub-band has the same false alarm rate and detection probability, the efficiency of wideband spectrum sensing mainly decided by the sub-band selection algorithm. In this section, we evaluate the O-D-UCB policy and compare with other policies which are employed to address sub-band selection problems. The detailed experimental setup and numerous quantitative analysis as well as visualization are also presented.

### 5.1. Datasets and Settings

For better modeling and analysis of the electromagnetic environment, the following assumptions were made during the simulation experiments. Firstly, the action of the policy has no effect on the reward change of per arm. Secondly, when the reward change, it will be not change again in *M* time slots, in this paper, we assume that *M = 5000*, it means that the probability distribution of reward will be stable for at least 5000 time slots. Then, the channels of wideband are independent channels; channel states do not affect each other. Lastly, the distributions of reward for arms are Bernoulli distributions, and it is independent identically distributed in time. Besides, we set the parameters of simulation data: Data size *N = 50,000*, the number of arms *K = 5*.

**Figure 4:**
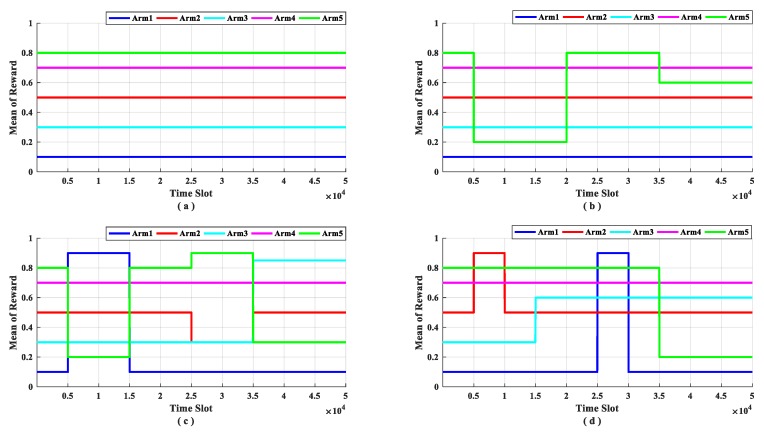
Datasets for different scenario. Y-label represents the average reward of each arm. (**a**) describes dataset a. (**b**) describes dataset b. (**c**) describes dataset c. (**d**) describes dataset d.

Based on this, according to the analysis of the variation law of the channel state probability distribution function, four data sets are designed based on the proposed four scenarios. Dataset a: Scenario 1 (stable scenario, Figure 4a); dataset b: Scenario 2 (scenario with one channel changes, Figure 4b); dataset c: Scenario 3 (scenario with multi arms change synchronized, Figure 4c) and dataset d: Scenario 4 (scenario with multi arms change out of synchronized, Figure 4d). In the rest of the paper, we use dataset a, dataset b, dataset c, and dataset d to represent different test scenarios respectively. 

As can be seen from Figure 4, in dataset a, the rewards of arms do not change and there is only one optimal arm in the whole time; in dataset b, there is only one arm changes its reward and the optimal arm changes over time, the reward of arm five changes three times, and the optimal arm changes tree times; in dataset c, there are multi arms change at the same time, the reward distribution of four arms changed, these changes occurred at four moments which leads to the optimal arm changes four times; and in dataset d, there are multi arms change out of sync, only arm two remained unchanged throughout the simulation, and the remaining arms changed at six moments, resulting in the optimal arm changes five times. This is summarized in Table 3. During the simulation experiment, we selected the accumulative pseudo-regret and the optimal arm utilization rate as performance indicators.

### 5.2. Parameters Selection

In this paper, as the parameters of the policy, we designed a variety of discount functions and exploration bonuses, so that the O-D-UCB policy can be adapted to different applications, such as sub-band selection, advertising recommendation. In order to study the performance of the O-D-UCB policy in the sub-band selection problem, we designed two sets of experiments to determine the most appropriate discount function and exploration bonus.

#### 5.2.1. Impact of Discount Function

**Figure 5:**
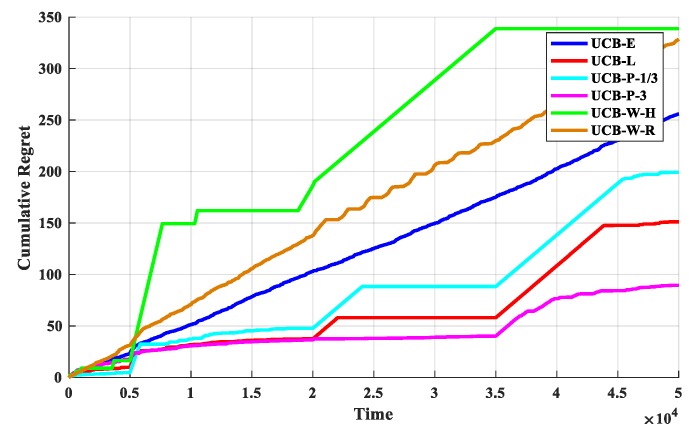
The cumulative regret for the O-D-UCB policies with different discount functions in dataset b.

**Figure 6:**
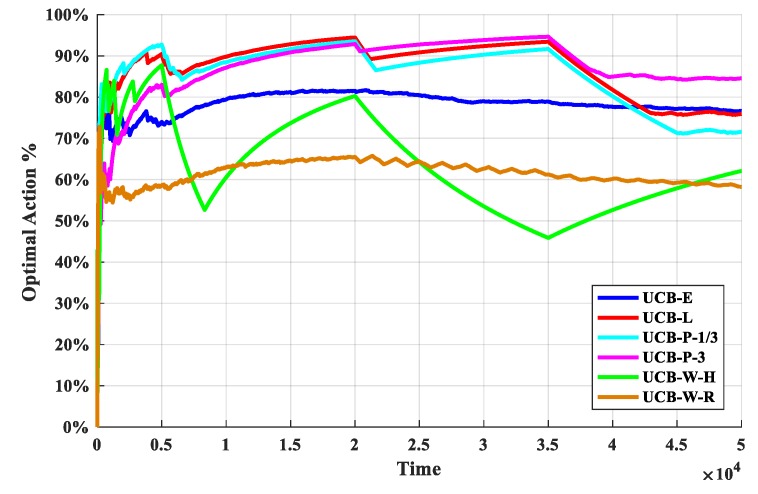
The optimal arm utilization rate for the O-D-UCB policies with different discount functions in dataset b.

The first set of experiments is used to determine the discount function. The O-D-UCB policies with different discount functions and the traditional exploration bonus are run in dataset b which is a typical scenario of the sub-band selection problem, and the results are shown in Figure 5 and Figure 6.

From Figure 5, it is clear that the UCB-P-3 policy has the best performance and the UCB-W-H policy has the worst performance. In additional, the performance gap between each policy is obvious, and the order of performance is: UCB-P-3 > UCB-L > UCB-P-1/3 > UCB-E > UCB-W-R > UCB-W-H. Then, the performances of UCB-E policy and UCB-W-R policy are stable, cumulative regret value is accumulated with a fixed slope. Furthermore, the cumulative regret value will increase with the increase of change points, and it will have a significant increase near the change point in generally, except UCB-E policy and UCB-W-R policy.

As we can see from Figure 6, after the start of the experiment, with the continuous exploration, the optimal arm utilization rate rapidly increases; when a certain stage is explored, the optimal arm utilization remains stable, and the exploration and exploitation reach a stable balance. With the arrival of the change point, the optimal arm utilization rate drops rapidly, but remain stable again soon. Therefore, in addition to the optimal arm rate can be used as a performance indicator, the change of the optimal arm utilization rate near the change point is also an important manifestation of the performance for the policies. 

We can see from Figure 6 that the optimal arm utilization rate of the UCB-P-3 policy is the highest throughout the experiment, especially after multiple change points. In additional, the optimal arm utilization rate of UCB-E policy and UCB-W-R policy remain at about 70% and 60%. Then, near the change point, the optimal arm utilization rate of the UCB-W-H policy fluctuate the most, followed by the UCB-W-H policy, and the least volatile is the UCB-P-3 algorithm. Finally, the optimal arm utilization of UCB-W-H policy will be difficult to remain stable, with the increasing of change points.

#### 5.2.2. Impact of Exploration Bonus

**Figure 7:**
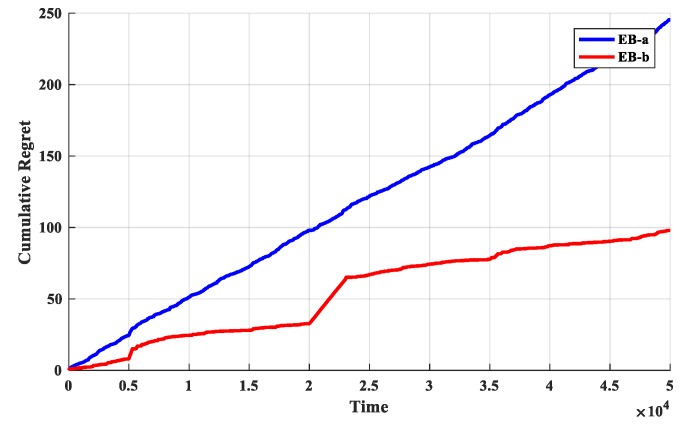
The cumulative regret of different policies for different scenarios.

**Figure 8:**
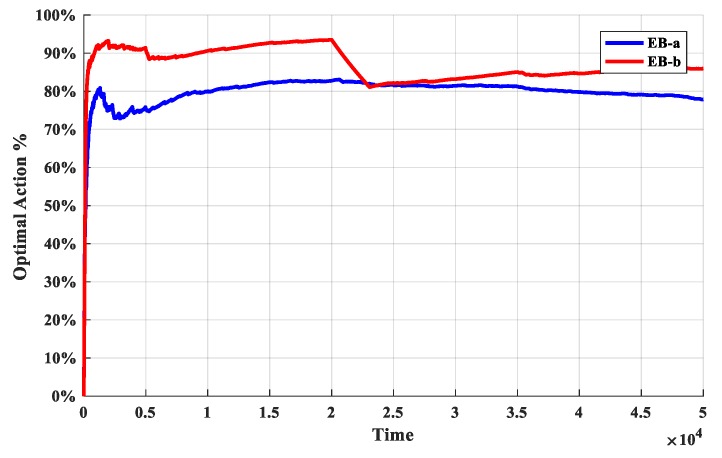
The optimal arm utilization rate of different policies for different scenarios.

The second set of experiments is used to determine the exploration bonus. The O-D-UCB policies with different exploration bonus are run in dataset b and the discount function is exponential function which described in Table 2. The simulation results are shown in Figure 7 and Figure 8.

It is obvious from Figure 7 that the cumulative regret values of the O-D-UCB policy with the EB-b is significantly less, but it grows rapidly near change point. In addition, the cumulative regret value curve of D-UCB with EB-a can be fitted as a straight line with steady rise, and it does not fluctuate obviously with the increase of change points.

It is obviously in Figure 8, the optimal arm utilization rate of D-UCB policy with EB-a remains stable and stable at around 70%, while that of D-UCB policy with EB-b fluctuates with change points. The fluctuation range at some change points is large, and the optimal arm utilization rate will continue to rise until it is stable after decreasing. Although the optimal arm utilization rate is fluctuating, in the case of D-UCB policy with EB-b, it is still higher than that of D-UCB policy with EB-a.

#### 5.2.3. Comparison

Through the above experiments, we find the optimal parameter combination of the O-D-UCB policy for sub-band selection problem, the discount function should be the power function with an exponent of three, whose expression is (11); and the exploration bonus should be the bonus based on the statistic variance, whose expression is (12). Therefore, the core expression of the O-D-UCB policy is shown in Table 4.

(11) $$f(x)={\left((N-x)/N\right)}^{3}$$

(12) $$B(x)=\sqrt{\left(X_{k}(t)-X_{k}{\left(t\right)}^{2})/N_{k}(t\right)}$$

In the simulation experiments, we compare the proposed optimal D-UCB policy against five other non-stationary policies: NRBE [40], WRBE [40], EXP3.S [23], SW-UCB [39], and D-UCB [39]. The parameter values for NRBE (using the notation in [40]) are γ=0.98 and $\tilde{n}=1700$, for WRBE the parameters are W=800. Using the notation in [23] the parameters for the EXP3.S policy are α=0.00005 and γ=0.0658. The parameter values for SW-UCB which using the notation in [39] are τ=1780 and ξ=0.6, for D-UCB the parameters are γ=0.9982 and ξ=0.15.

**Figure 9:**
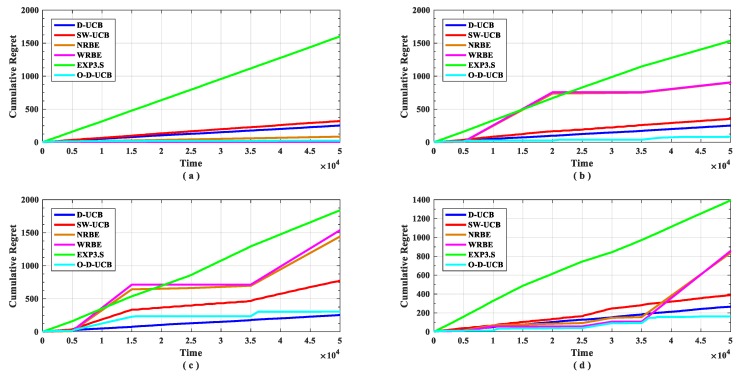
The cumulative regret of each policy under different datasets. (**a**) describes dataset a. (**b**) describes dataset b. (**c**) describes dataset c. (**d**) describes dataset d.

**Figure 10:**
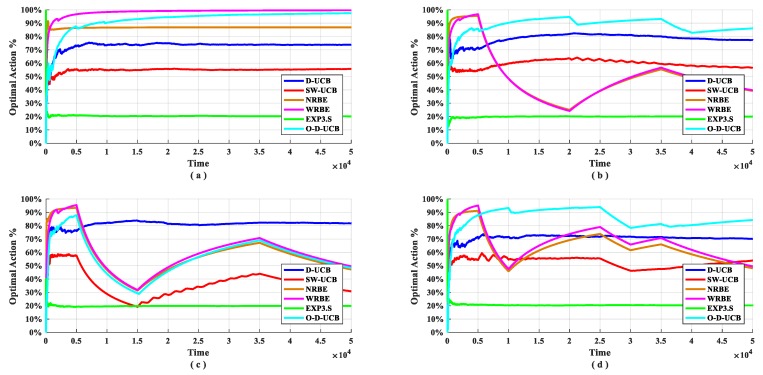
The optimal arm utilization rate of each policy under different datasets. (**a**) describes dataset a. (**b**) describes dataset b. (**c**) describes dataset c. (**d**) describes dataset d.

The performance of the O-D-UCB policy with other policies in different data sets is shown in the Figure 9 and Figure 10.

As shown in Figure 9, in dataset a (Figure 9a), the policy with the lowest cumulative regret is the WRBE policy, but the gap of cumulative regret between each policy is not large except the EXP3.S policy. In non-stationary scenarios (Figure 9b–d), the policy with the lowest cumulative regret is the O-D-UCB policy, except in dataset c (Figure 9c). In dataset c (Figure 9c), the D-UCB policy has the lowest cumulative regret, but the performance gap with the O-D-UCB policy is small. In all data sets, the performance of the D-UCB algorithm remains stable; after the simulation time, the accumulated regret value is stable at around 250. EXP3.S has the worst performance in all data sets. When there is a change point, the optimal D-UCB policy can converge quickly at each data set, and some policies cannot converge at some data set. When the change point number is small, the NRBE and WRBE policies can converge quickly, as the change point increases, the convergence period is significantly longer, and the convergence value increases significantly, in the end, it does not converge at the dataset c (Figure 9c) and dataset d (Figure 9d).

We can get from Figure 10, in the stationary scenario (Figure 10a), the optimal arm usage of O-D-UCB policy and WRBE policy can reach more than 90%, and the NRBE policy can maintained near 85%, the D-UCB policy is maintained at around 75%, and the SW-UCB policy is slightly higher than 50%, EXP3.S is lowest, it only reached 20%. In all non-stationary scenarios (Figure 10b–d), the optimal arm usage of D-UCB policy is kept at around 75%, while the O-D-UCB policy can reach 80% or more in the dataset b and dataset d, and in dataset c (Figure 10c), the fluctuation range is large, resulting in a lower optimal arm usage rate. In Figure 10, a more interesting phenomenon is that the optimal arm usage of the EXP3.S policy is kept at around 20% in all data sets, which is the same as the probability of randomly selecting the optimal arm.

## 6. Discussion

### 6.1. Parameters

The discount function represents the use of historical samples by the policy; the exploration bonus represents the tradeoff of the exploration and exploitation. In order to get better performance, these two parameters need to be properly matched according to the application requirements. Such as the discount function *y = ((N − x/N)^3^* which we selected in this paper has a higher degree of utilization of recent samples and a lower degree of utilization of historical samples. The other discount function *y = ((N − x/N)^1/3^* is different, it has a high degree of utilization for all samples, but the samples at the beginning of the experiment (Figure 3). It can be seen that these two discount functions are adapted to different application scenarios: The discount function *y = ((N − x/N)^3^* is adapted to a scene where the change is frequent, and the policy only needs to consider the recent samples; the discount function *y = ((N − x/N)^1/3^* is adapted to a scene with little change, and the policy need to consider all of the historical samples. Therefore, the discount function *y = ((N − x/N)^3^* is suitable for sub-band selection problem, this is the same choice of the experiments (Figure 4 and Figure 5).

### 6.2. Performance

It can be seen from Figure 9 and Figure 10 that the O-D-UCB policy which proposed in this paper can obtain a better sub-band sensing sequence in a dynamic environment by comparing with other commonly used policies [23,39,40] in different data sets through simulation experiment, especially in dataset b and dataset d. In dataset c, the performance of the O-D-UCB policy is slightly lower than that of the D-UCB policy. 

Here, we focus on the reasons for the performance degradation of the policy in dataset c. Before the first change point, since exploration and exploitation form stability, the optimal arm is the mostly used, and the worst arm is rarely chosen. When the change occurs, the original optimal arm becomes the worst arm, and the original worst arm becomes the optimal arm. It is difficult to find the optimal arm after the change in a short time, therefore, after the first change point, the optimal arm usage rate drops rapidly and it is difficult to stabilize. In this instance, the second change point swaps the optimal arm and the worst arm, so after the second change point, the optimal arm usage rate rises sharply and the third change point does not change the position of the optimal arm. Therefore, there is no influence on the trend of the optimal arm usage rate. At the fourth change point, the optimal arm becomes the second worst arm, and the second worst arm becomes the optimal arm. So, the optimal D-UCB policy takes a long time to find the optimal arm and the optimal arm usage rate drops rapidly.

According to the above analysis, we need to increase the exploration of the policy so that the policy can quickly find the optimal arm in the dramatic change.

### 6.3. Cost

Since the policy proposed in this paper is based on the traditional D-UCB policy, so we only analyzed the cost of D-UCB policy and the O-D-UCB policy, the core calculation expressions of two policies are described in the Table 4. We can know that the computation complexity of exponential function is $O(2^{n})$, where *n* is the simulation time slot; and the computation complexity of power function is $O(n^{3})$, where *n* is the simulation time slot. So we can get that the computation complexity of exploitation function of these two policies is respectively $O(2^{n})$ and $O(n^{3})$: $T(X_{k}(t)|D-\mathrm{UCB})=O(2^{n})$, $T(X_{k}(t)|\mathrm{optimized}D-\mathrm{UCB})=O(n^{3})$. On this basis, we further analyze the computational complexity of exploration reward, for D-UCB policy, the computation complexity of exploration reward is: $T(B_{k}(t))=\sum_{j=1}^{K}O(2^{n})=K*O(2^{n})=O(2^{n})$; the computation complexity of exploration reward for O-D-UCB policy is: $T(B_{k}(t))=O(n^{3})*O(n^{3})=O(n^{6})$. The computation complexity of these two policies is the sum of the complexity of the exploitation function and exploration reward. So the computation complexity of D-UCB policy is: $T(U_{k}(t))=T(X_{k}(t))+T(B_{k}(t))=O(2^{n})$; the computation complexity of O-D-UCB policy is: $T(U_{k}(t))=T(X_{k}(t))+T(B_{k}(t))=O(n^{3})+O(n^{6})=O(n^{6})$. We can get that the computation complexity of the O-D-UCB policy is smaller than D-UCB policy ($O(n^{6})<O(2^{n})$), which are summarized in Table 5.

## 7. Conclusions

In this paper, we propose an O-D-UCB policy for NS-MAB model for sub-band selection problem of wideband spectrum sensing. Through multiple experiments, the optimal discount function and exploration bonus of D-UCB policy were found. Based on this, we refine the parameters in the O-D-UCB policy for sub-band selection problem. Extensive experiments on four data sets indicate that the O-D-UCB policy is effective and efficient enough for sub-band selection problems, especially in the high dynamic electromagnetic environment. Furthermore, the O-D-UCB policy is adequate to NS-MAB model when there is no prior knowledge of the reward of each arm. However, we only studied the design methods of discount function and exploration bonus from experiment. It is also indicated that the O-D-UCB policy can improve the decision performance of the NS-MAB problems, but the theoretical basis of the policy will be left as further open research. Besides, in future research, we also need to solve the problem of performance improvement in the scene with the rewards of multi arms change synchronized.

## Figures and Tables

**Table 1:** The summary of policies for MAB problems.

	Stationary Independent Reward	Non-stationary Independent Reward	
**Randomized**	ε-greedy cluster EXP3 cluster Soft-max	ε-greedy cluster EXP3.S	
**UCB-based**	UCB RUCB KL-UCb	D-UCB SW-UCB NRBE WRBE	
**Recency-based**	RBE RBERC		
	
**Deterministic**	DSEE		
	

Discounted upper confidence bound (D-UCB); sliding window upper confidence bound (SW-UCB); non-stationary recency-based exploration (NRBE); and windowed recency-based exploration (WRBE).

**Table 2:** The correspondence between the abbreviation of policy and the discount function expression.

Abbreviation of Policy	Discount Function Expression
UCB-E	*y = 0.9982^x^*
UCB-L	*y = (N − x)/N*
UCB-P-1/3	*y = ((N − x)/N)^1/3^*
UCB-P-3	*y = ((N − x)/N)^3^*
UCB-W-H	y = (1 + cos(pi*x/N))/2
UCB-W-R	*y = R(x)*

**Table 3:** The characteristics of datasets.

Datasets	The Number of Change Points	The Number of Optimal Arm Change
**dataset a**	0	0
**dataset b**	3	3
**dataset c**	4	4
**dataset a**	6	5

**Table 4:** D-UCB and O-D-UCB corresponding quantities.

D-UCB Policy	O-D-UCB Policy
$U_{k}(t):=X_{k}(t)+B_{k}(t)$	$U_{k}(t):=X_{k}(t)+B_{k}(t)$
$X_{k}(t)=\sum_{s=1}^{t}\gamma^{t-s}X_{s}^{k}1_{i_{s}=i}/N_{k}(t)$	$X_{k}(t)=\sum_{s=1}^{t}{\left((N-s)/N\right)}^{3}X_{s}^{k}1_{i_{s}=i}/N_{k}(t)$
$N_{k}(t)=\sum_{s=1}^{t}\gamma^{t-s}1_{i_{s}=i}$	$N_{k}(t)=\sum_{s=1}^{t}{\left((N-s)/N\right)}^{3}1_{i_{s}=i}$
$B_{k}(t)=2B\sqrt{\xi \log(\sum_{j=1}^{K}N_{j}(t))/N_{k}(t)}$	$B_{k}(t)=\sqrt{\left(X_{k}(t)-X_{k}{\left(t\right)}^{2})/N_{k}(t\right)}$

**Table 5:** The computation complexity of D-UCB policy and O-D-UCB policy.

Computation Complexity	D-UCB Policy	O-D-UCB Policy
$T(X_{k}(t))$	$O(2^{n})$	$O(n^{3})$
$T(B_{k}(t))$	$O(2^{n})$	$O(n^{6})$
$T(U_{k}(t))$	$T(X_{k}(t))+T(B_{k}(t))=O(2^{n})$	$T(X_{k}(t))+T(B_{k}(t))=O(n^{6})$

## References

[1] Liu X., Jia M., Zhang X., Lu W. (2019). A Novel Multichannel Internet of Things Based on Dynamic Spectrum Sharing in 5G Communication. IEEE Internet Things J..

[2] Awin F.A., Alginahi Y.M., Abdel-Raheem E., Tepe K. (2019). Technical Issues on Cognitive Radio-Based Internet of Things Systems: A Survey. IEEE Access.

[3] Amjad M., Rehmani M.H., Mao S. (2018). Wireless Multimedia Cognitive Radio Networks: A Comprehensive Survey. IEEE Commun. Surv. Tutor..

[4] Liu C., Wang H., Zhang J., He Z. (2018). Wideband Spectrum Sensing Based on Single-Channel Sub-Nyquist Sampling for Cognitive Radio. Sensors.

[5] Arjoune Y., Kaabouch N. (2019). A Comprehensive Survey on Spectrum Sensing in Cognitive Radio Networks: Recent Advances, New Challenges, and Future Research Directions. Sensors.

[6] Hamdaoui B., Khalfi B., Guizani M. (2018). Compressed Wideband Spectrum Sensing: Concept, Challenges, and Enablers. IEEE Commun. Mag..

[7] Jayaweera S.K. (2014). Signal Processing for Cognitive Radios.

[8] Liu X., Jia M., Na Z., Lu W., Li F. (2018). Multi-Modal Cooperative Spectrum Sensing Based on Dempster-Shafer Fusion in 5G-Based Cognitive Radio. IEEE Access.

[9] Awin F., Abdel-Raheem E., Tepe K. (2019). Blind Spectrum Sensing Approaches for Interweaved Cognitive Radio System: A Tutorial and Short Course. IEEE Commun. Surv. Tutor..

[10] Chen Y., Su S., Yin H., Guo X., Zuo Z., Wei J., Zhang L. (2019). Optimized Non-Cooperative Spectrum Sensing Algorithm in Cognitive Wireless Sensor Networks. Sensors.

[11] Liu X., Li F., Na Z. (2017). Optimal Resource Allocation in Simultaneous Cooperative Spectrum Sensing and Energy Harvesting for Multichannel Cognitive Radio. IEEE Access.

[12] Oksanen J., Koivunen V. (2015). An Order Optimal Policy for Exploiting Idle Spectrum in Cognitive Radio Networks. IEEE Trans. Signal Process..

[13] Oksanen J. (2016). Machine Learning Methods for Spectrum Exploration and Exploitation.

[14] Thompson W.R. (1933). On the Likelihood that One Unknown Probability Exceeds Another in View of the Evidence of Two Samples. Biometrika.

[15] Robbins H. (1952). Some aspects of the sequential design of experiments. Bull. Am. Math. Soc..

[16] Auer P., Cesa-Bianchi N., Freund Y., Schapire R.E. Gambling in a Rigged Casino: The Adversarial Multi-Armed Bandit Problem. Proceedings of the IEEE 36th Annual Foundations of Computer Science.

[17] Watkins C.J. (1989). Learning from Delayed Rewards. Ph.D. Thesis.

[18] Tran-Thanh L., Chapman A., de Cote E.M., Rogers A., Jennings N.R. Epsilon–First Policies for Budget–Limited Multi-Armed Bandits. Proceedings of the Twenty-Fourth AAAI Conference on Artificial Intelligence.

[19] Tokic M., Palm G. (2011). Value-Difference Based Exploration: Adaptive Control between Epsilon-Greedy and Softmax.

[20] Cesa-Bianchi N., Fischer P. (1998). Finite-Time Regret Bounds for the Multiarmed Bandit Problem. Proceedings of the ICML.

[21] Vermorel J., Mohri M., Gama J., Camacho R., Brazdil P.B., Jorge A.M., Torgo L. (2005). Multi-armed Bandit Algorithms and Empirical Evaluation. Proceedings of the Machine Learning: ECML 2005.

[22] Luce R.D. (1959). Individual Choice Behavior.

[23] Auer P., Cesa-Bianchi N., Freund Y., Schapire R. (2002). The Nonstochastic Multiarmed Bandit Problem. SIAM J. Comput..

[24] Bubeck S., Slivkins A. The Best of Both Worlds: Stochastic and Adversarial Bandits. Proceedings of the Conference on Learning Theory.

[25] Auer P., Cesa-Bianchi N., Fischer P. (2002). Finite-time Analysis of the Multiarmed Bandit Problem. Mach. Lear..

[26] Agrawal R. (1995). Sample mean based index policies by O(log n) regret for the multi-armed bandit problem. Adv. Appl. Probab..

[27] Bubeck S. (2010). Bandits Games and Clustering Foundations. Ph.D. Thesis.

[28] Garivier A., Cappé O. The KL-UCB Algorithm for Bounded Stochastic Bandits and Beyond. Proceedings of the 24th Annual Conference on Learning Theory.

[29] Li L., Chu W., Langford J., Schapire R.E. A Contextual-bandit Approach to Personalized News Article Recommendation. Proceedings of the 19th International Conference on World Wide Web.

[30] Tekin C., Liu M. (2012). Online Learning of Rested and Restless Bandits. IEEE Trans. Inf. Theory.

[31] Liu H., Liu K., Zhao Q. (2013). Learning in a Changing World: Restless Multiarmed Bandit With Unknown Dynamics. IEEE Trans. Inf. Theory.

[32] Ortner R., Ryabko D., Auer P., Munos R., Bshouty N.H., Stoltz G., Vayatis N., Zeugmann T. (2012). Regret Bounds for Restless Markov Bandits. Proceedings of the Algorithmic Learning Theory.

[33] Bubeck S., Cohen M., Li Y. Sparsity, Variance and Curvature in Multi-Armed Bandits. Proceedings of the Algorithmic Learning Theory.

[34] Jun K., Jamieson K., Nowak R., Zhu X. Top Arm Identification in Multi-Armed Bandits with Batch Arm Pulls. Proceedings of the Artificial Intelligence and Statistics.

[35] Liu F., Lee J., Shroff N. A Change-Detection Based Framework for Piecewise-Stationary Multi-Armed Bandit Problem. Proceedings of the Thirty-Second AAAI Conference on Artificial Intelligence.

[36] Pang K., Dong M., Wu Y., Hospedales T. Dynamic Ensemble Active Learning: A Non-Stationary Bandit with Expert Advice. Proceedings of the 24th International Conference on Pattern Recognition (ICPR).

[37] Kocsis L., Szepesvári C. Discounted Ucb. Proceedings of the 2nd PASCAL Challenges Workshop.

[38] Saito K., Notsu A., Honda K. Discounted UCB1-tuned for Q-learning. Proceedings of the Joint 7th International Conference on Soft Computing and Intelligent Systems (SCIS) and 15th International Symposium on Advanced Intelligent Systems (ISIS).

[39] Garivier A., Moulines E., Kivinen J., Szepesvári C., Ukkonen E., Zeugmann T. (2011). On Upper-Confidence Bound Policies for Switching Bandit Problems. Proceedings of the Algorithmic Learning Theory.

[40] Oksanen J., Koivunen V. Learning Spectrum Opportunities in Non-Stationary Radio Environments. Proceedings of the IEEE International Conference on Acoustics, Speech and Signal Processing (ICASSP).

[41] Alaya-Feki A.B., Moulines E., LeCornec A. Dynamic spectrum access with non-stationary Multi-Armed Bandit. Proceedings of the IEEE 9th Workshop on Signal Processing Advances in Wireless Communications.

[42] Shedden K. (2013). Binomial Confidence Intervals.

